# Genotyping of Salmon Gill Poxvirus Reveals One Main Predominant Lineage in Europe, Featuring Fjord- and Fish Farm-Specific Sub-Lineages

**DOI:** 10.3389/fmicb.2020.01071

**Published:** 2020-05-29

**Authors:** Snorre Gulla, Torstein Tengs, Saima Nasrin Mohammad, Mona Gjessing, Åse Helen Garseth, Karoline Sveinsson, Torfinn Moldal, Petra E. Petersen, Brit Tørud, Ole Bendik Dale, Maria K. Dahle

**Affiliations:** ^1^Norwegian Veterinary Institute, Oslo, Norway; ^2^Department of Molecular Biology, Norwegian Institute of Public Health, Oslo, Norway; ^3^Faroese Food and Veterinary Authority, Tórshavn, Faroe Islands; ^4^The Norwegian College of Fishery Science, Faculty of Biosciences, Fisheries and Economics, UiT – The Arctic University of Norway, Tromsø, Norway

**Keywords:** salmon gill poxvirus (SGPV), MLVA, VNTR, microsatellite, genotyping, fish disease, Atlantic salmon (*Salmo salar*), aquaculture

## Abstract

Salmon gill poxvirus (SGPV) can cause serious gill disease in Atlantic salmon (*Salmo salar* L.) and represents a significant problem to aquaculture industries in Northern Europe. Here, a single-tube multi-locus variable-number tandem-repeat (VNTR) analysis (MLVA) genotyping assay, targeting eight VNTR loci, was developed for studying the epizootiology of SGPV. Through MLVA typing of SGPV positive samples from 180 farmed and wild Atlantic salmon in Northern Europe, the first molecular population study of this virus was undertaken. Comparison of resulting MLVA profiles by cluster analysis revealed considerable micro-diversity, while only a limited degree of specific clustering by country of origin could be observed, and no clustering relating to the severity of disease outbreaks. Phylogenetic analysis, based on genomic data from six SGPV specimens (three Norwegian, one Scottish, one Faroese and one Canadian), complemented and corroborated MLVA by pointing to a marked transatlantic divide in the species, with one main, relatively conserved, SGPV lineage as predominant in Europe. Within certain fjord systems and individual freshwater salmon smolt farms in Norway, however, discrete MLVA clustering patterns that prevailed over time were observed, likely reflecting local predominance of specific SGPV sub-lineages. MLVA typing was also used to refute two suspected instances of vertical SGPV transmission from salmon broodstock to offspring, and to confirm a failed disinfection attempt in one farm. These novel insights into the previously undocumented population structure of SGPV provide important clues, e.g., regarding the mechanisms underlying spread and recurrence of the virus amongst wild and farmed salmon populations, but so far no indications of more or less virulent SGPV sub-lineages have been found. The MLVA scheme represents a highly sensitive genotyping tool particularly well suited for illuminating SGPV infection routes, and adds to the relatively low number of MLVA protocols that have so far been published for viral species. Typing is reasonably inexpensive, with a moderate technological requirement, and may be completed within a single working day. Resulting MLVA profiles can be readily shared and compared across laboratories, facilitating rapid placement of samples in an international ezpizootiological context.

## Introduction

While historic and ongoing expansions of industrialized aquaculture activities worldwide are among the most important initiatives for feeding a growing global population, these industries have suffered significant setbacks, e.g., due to infectious fish diseases. In Norway, the world’s largest producer of farmed Atlantic salmon (*Salmo salar* L.), minimizing disease-related mortalities has been a priority, and farmed salmon today are routinely and efficiently vaccinated against an array of bacterial pathogens and a few viral agents (Brudeseth et al., 2013). Nevertheless, several viruses still pose significant threats, mainly due to missing vaccines or low vaccine efficiency (Robertsen, 2011).

Members of the *Poxviridae* family are large enveloped double-stranded DNA viruses (200–300 nm) that replicate in the cytoplasm of infected cells (Tolonen et al., 2001). An unidentified poxvirus was suspected to cause acute gill disease in farmed Atlantic salmon juveniles in Norway as early as during the 1990s, but it was not until 2008 that the first report on viral particles resembling a poxvirus in salmon gills, observed by transmission electron microscopy, was published (Nylund et al., 2008). The virus was named salmon gill poxvirus (SGPV), but another seven years, and the advent of next-generation sequencing, passed before a breakthrough was made in 2015 when the genome was sequenced (Gjessing et al., 2015). The SGPV genome (GenBank accession no. KT159937) consists of a single large (∼242 kbp) linear dsDNA segment, which phylogenetically represents the deepest recognized branch within the *Chordopoxvirinae* subfamily (Gjessing et al., 2015). Its characterization enabled the development of novel diagnostic approaches, including qPCR assays and antibodies for immunohistochemistry, which in turn allowed confirmation of a close association between SGPV presence/localization and the typical gill pathology seen in disease outbreaks (Gjessing et al., 2015). A challenge model proving causality is currently under development (unpublished data).

Salmon gill poxvirus is now recognized as a widespread virus in Norwegian salmon farming, where it commonly causes recurring acute disease outbreaks of varying severity, although subclinical SGPV detections are also regularly made. Clinical outbreaks are often associated with complex gill disease concurrently with a diverse range of cellular organisms, including, e.g., *Ichthyobodo* spp., *Paramoeba perurans*, *Saprolegnia* spp., *Desmozoon lepeophtherii*, and *Ca*. Branchiomonas cysticola (Gjessing et al., 2017; Hjeltnes et al., 2019). In Norway, screening for SGPV by qPCR has further revealed its relatively widespread presence in wild Atlantic salmon spawners captured in Norwegian rivers (Garseth et al., 2018; Gåsnes et al., 2019), and the virus has also been detected in farmed salmon in other northern European countries, including Scotland and The Faroe Islands (Gjessing et al., 2018). Moreover, an SGPV variant strain was also recently reported and characterized from Atlantic salmon in Eastern Canada (Leblanc et al., 2019).

Despite the wide distribution of SGPV, published data on the extent of genetic diversity within the viral population(s) is lacking. For instance, the highly variable clinical presentations reported in relation to detections could conceivably reflect the existence of more or less virulent strains of the virus. Unfortunately however, while the Canadian strain reportedly replicated in cell cultures (Leblanc et al., 2019), repeated attempts of viral cultivation and isolation from European SGPV samples have not yet been successful, thus limiting further molecular and functional investigations. High-throughput (e.g., Illumina) sequencing performed directly on infected gill samples was therefore necessary for the genomic characterization of SGPV, but as this approach is relatively expensive and laborious, it is unsuitable for routine use on large numbers of samples.

Multi-locus variable-number tandem-repeat analysis (MLVA) constitutes a molecular genotyping method particularly well suited for investigating close evolutionary relationships between conspecific biological specimens. The technique exploits a pre-defined selection of hypervariable genomic loci, commonly named variable-number tandem-repeats (VNTR) or mini-/microsatellites, which, over generations, are subject to frequent successive changes in size. The approach is fast, inexpensive and produces readily transferrable data, while providing an epidemiological resolution in some cases matching that of whole genome sequencing (Eyre et al., 2013; Limmathurotsakul et al., 2014; Rashid et al., 2016). Historically, MLVA has been extensively used for the epidemiological investigation of bacterial pathogens (e.g., Malorny et al., 2008; Li Y. et al., 2009; Gulla et al., 2018), but VNTR regions have also been shown to account for a major proportion of the genetic polymorphism within some large viruses (Houng et al., 2009; Avarre et al., 2011). A limited number of VNTR-based typing protocols have been developed for viral species, in some cases awarding significantly improved resolution when compared to genotyping by, e.g., restriction fragment length polymorphisms or single-/multi-gene sequencing (Deback et al., 2009; Houng et al., 2009; Avarre et al., 2011; Burrel et al., 2013).

In light of the increasingly recognized significance of SGPV in salmon farming internationally, and the potential related risks for wild salmon populations, there is an acute need for establishing expedient, high-resolution genotyping tools enabling epizootiological investigations. The very large genome of this virus, in combination with the apparent multitude of repetitive DNA regions contained within it (Gjessing et al., 2015), furthermore makes SGPV a fitting candidate for MLVA typing. The primary aim of the present study was therefore to establish such an assay, and to conduct an MLVA genotyping survey highlighting SGPV population dynamics.

## Materials and Methods

### High-Throughput Sequencing and Contig Assembly

To generate additional SGPV genome information, one gill sample from wild Norwegian Atlantic salmon, and three additional gill samples from farmed Atlantic salmon (Norwegian, Faroese and Scottish) (see Supplementary Table S1), all displaying relatively high SGPV loads (Ct-values < 19; qPCR as described by Gjessing et al., 2015), were selected for sequencing. Total genomic DNA was extracted by use of a DNeasy Blood & Tissue Kit (Qiagen), according to the manufacturer’s recommendations, and subjected to quantification and quality control by use of a Qubit fluorometer with the Qubit dsDNA BR Assay (Life Technologies). Following library preparation using TruSeq PCR-free prep (Illumina Inc., San Diego, CA, United States), paired-end 150 bp sequencing was performed on an Illumina HiSeq sequencer (Illumina Inc., San Diego, CA, United States). Resulting raw reads were trimmed with Trimmomatic (Bolger et al., 2014), employing recommended settings for paired-end reads, and then aligned with the Atlantic salmon genome (assembly ICSASG_v2; Lien et al., 2016) using the Burrows-Wheeler Aligner package (Li and Durbin, 2009). Matching reads were removed with SAMtools (Li H. et al., 2009), while remaining reads were *de novo* assembled using SPAdes (Bankevich et al., 2012; Nurk et al., 2013) with default parameters. Contigs overlapping with the genome of SGPV strain 2012-04-F277-L3G (GenBank accession no. KT159937) were identified using nucleotide BLAST (Altschul et al., 1990).

### Phylogenetic Analysis

The amino acid sequences of two genes (major capsid protein and DNA-directed RNA polymerase subunit beta) were acquired from various published *Poxviridae* genomes (Leblanc et al., 2019), and from the SGPV contig sets described above. Using Clustal X (Larkin et al., 2007) with default settings, alignments were made for both genes individually, and for their concatenated 1613-1760 amino acid sequence. Maximum Likelihood trees were made from the alignments using PhyML 3.0 (Guindon et al., 2010) with SMS (Lefort et al., 2017) activated, and with branch support estimated by the aLRT SH-like method (Anisimova and Gascuel, 2006). The resulting trees were aesthetically modified using MEGA-X (Kumar et al., 2018).

### Fish Sample Collection for Genotyping Survey

**FIGURE 1:**
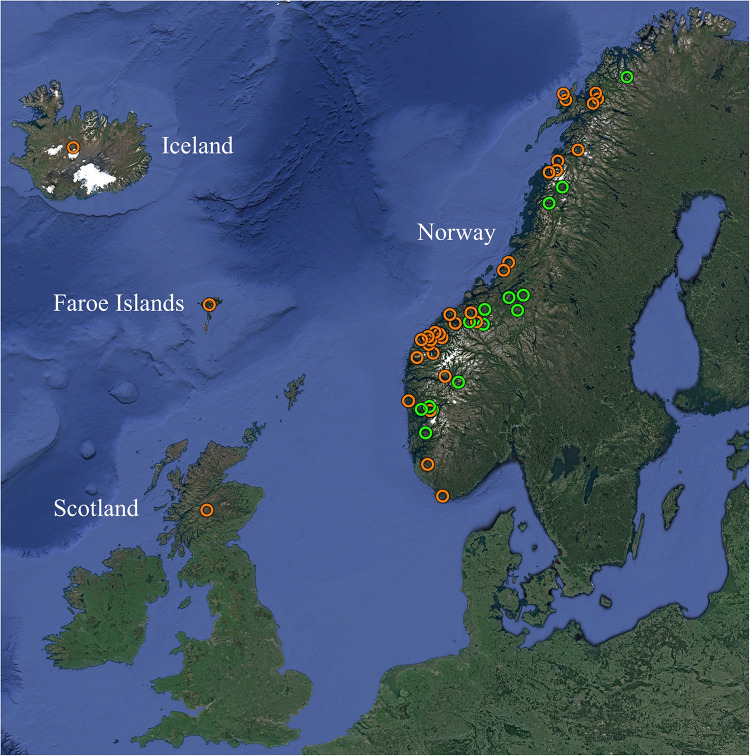
Overview of the geographic origins of SGPV positive samples from farmed (orange) and wild (green) Atlantic salmon MLVA typed in the present study. See Table 1 and Supplementary Table S1 for further information. Map created with Google Earth (Map data/imagery: IBCAO, Landsat/Copernicus, SIO, NOAA, U.S. Navy, NGA, GEBCO, U.S. Geological Survey).

A total of 310 gill samples were initially compiled, consisting primarily of diagnostic samples harvested from farmed and wild Atlantic salmon over multiple years in four European countries (Table 1, Supplementary Table S1, and Figure 1). All samples had tested positive for SGPV by use of a previously published qPCR assay (Gjessing et al., 2015), with reported Ct-values ranging from 16 to 37. Total gDNA was extracted as described above and subsequently used as template for downstream applications, or stored at −20°C until such use.

**TABLE 1:** From the 310 Atlantic salmon gill samples testing positive for SGPV by qPCR, the table shows the origins of the 180 samples successfully MLVA typed in the present study.

**Country**	**Environment**	**Physiological stage**	**Sampling timespan**	**No. sites sampled**	**No. samples MLVA typed**
Norway	Freshwater farms	Pre-smolts	1990s-2019	21	109
	Seawater farms	Post-smolts	2015–2019	8	12
	Rivers	Wild spawners	2009–2015	14	41
Scotland	Freshwater farms	Pre-smolts	2017–2019	2	8
	Seawater farms	Post-smolts	2019	2	2
Faroe Islands	Freshwater farms	Pre-smolts	2017	1	2
Iceland	Freshwater farms	Pre-smolts	2019	1	6

*See Supplementary Table S1 for detailed information on individual samples. The 130 samples excluded due to either lacking VNTR amplicons by PCR or ambiguous MLVA profiles (see section “Results,” Figure 3, and Supplementary Figure S2) are not considered here.*

### Identification of Informative VNTR Loci

The five European SGPV genomic contig sets (one previously published and four generated during the present study) were all subjected to analysis with Tandem Repeats Finder v4 (Benson, 1999). The subsequent process of selecting putatively repetitive loci for MLVA inclusion involved a geographically diverse collection of twelve SGPV positive samples being subjected to singleplex PCR and Sanger sequencing for evaluating the suitability of each candidate VNTR locus individually. VNTR selection criteria were (i) ubiquitous occurrence in the tested SGPV samples, (ii) repeat unit size uniformity, (iii) a degree of inter-strain copy number variation, and (iv) sufficiently conserved flanking regions. While minor repeat unit sequence heterogeneity was tolerated, conservation of repeat unit size was set as an absolute requirement to allow precise calling of repeat numbers by capillary electrophoresis. In accordance with suggested guidelines (Nadon et al., 2013), the selected VNTR loci were annotated according to their position (closest kbp) within the genome of SGPV strain 2012-04-F277-L3G (Supplementary Table S2).

### Multiplex PCR and Capillary Electrophoresis

A multiplex PCR assay was established involving 16 primers pairwise targeting each of the eight VNTR loci (Supplementary Table S3). Primers were designed using Geneious R7 (Biomatters), and checked for formation of secondary structures with Multiple Primer Analyzer (ThermoFisher Scientific) and for SGPV specificity by performing BLAST searches against the NCBI nucleotide collection. For each primer pair (Applied Biosystems), either of the forward- or reverse primer was labeled at the 5’ end with either of four fluorescent dyes (6FAM, VIC, NED, or PET). Particular care was taken to avoid amplicon size overlap between loci labeled with identical dyes.

Multiplex PCR mixtures contained 10 μl 2× Multiplex PCR master mix (Qiagen), 0.2 μM of each primer, 1 μl DNA template, and a volume of RNase-free water amounting to a total reaction volume of 20 μl. Subsequent PCR involved (i) 5 min at 95°C (ii) 30 cycles of 0.5 min at 95°C, 3 min at 60°C, and 1 min at 72°C, and (iii) 45 min at 68°C, followed by cooling to 4°C indefinitely. PCR products were verified by gel electrophoresis (as shown in Supplementary Figure S1) and then diluted 1:8 (vol/vol) in Milli-Q water. From the dilutions, 1 μl was added to 8.5 μl Hi-Di formamide (Applied Biosystems) and 0.5 μl GeneScan 600 LIZ dye size standard v2.0 (Applied Biosystems). Samples were then denatured for 5 min at 95°C prior to capillary electrophoresis on an Avant 3500xl Genetic Analyser (Applied Biosystems) utilizing POP-7 polymer (Applied Biosystems) and the following settings: 5 s injections at 1.6 kV (32 V/cm), and a 32 min run time at 15 kV (300 V/cm) and 60°C.

### VNTR Amplicon Size Calling, Correction, and MLVA Profiling

Following capillary electrophoresis, electrophoretic peaks were size called with GeneMapper 5 (Applied Biosystems) and assigned to appropriate VNTR loci according to fluorescent labeling and size. Samples displaying multiple ambiguous signals were excluded at this stage unless all secondary peaks displayed less than half the signal intensity of the primary peaks (see section “Results” and Supplementary Figure S2 for further details).

VNTR amplicon sizes called by capillary electrophoresis displayed some disagreement with Sanger sequence sizes, a phenomenon attributed to biased mobility patterns of amplicons in capillary electrophoresis machines (Lista et al., 2006; Pasqualotto et al., 2007). These size calls were therefore subjected to locus-specific corrections and converted to VNTR repeat counts as previously described (Gulla et al., 2018). Briefly, for each VNTR locus in 9–12 SGPV specimens displaying a range of alleles, linear regression was used to compare capillary electrophoresis- and Sanger sequence size estimates. This allowed calculation of line-of-best-fit equations, which were employed for correcting capillary electrophoresis size calls (Supplementary Figure S3). Each SGPV specimen thus received an eight-digit integer string (MLVA profile) reflecting the number of whole repeats determined at each VNTR locus. An exception was however made for locus SGPV_143, wherein some variants proved to carry a 6 bp insertion directly downstream of the repeat region. Having a repeat size of 13 bp, the SGPV_143 repeat count for alleles harboring this insertion was thus instead rounded upward to the nearest half. Absent capillary electrophoresis peaks in any locus (only relevant in two specimens for SGPV_67) were assigned a repeat count of 0.

### Allelic Diversity and Statistical Evaluation

The discriminatory capacity of the studied VNTR loci, individually and in combination, was estimated through calculation of Simpson’s indexes of diversity (Simpson, 1949) on the basis of observed allelic diversities. LIAN version 3.7 (Haubold and Hudson, 2000), employing the Monte Carlo-model with 10,000 iterations, was used for detecting possible linkage disequilibrium occurring amongst the loci. Only single representatives for each MLVA profile were included for LIAN analysis.

As outlined in the Supplementary Datasheet, the statistical likelihood of VNTR matches occurring by chance between random samples (in relation to dataset size) was estimated according to the allelic diversity observed in the VNTRs (see section “Results” and Table 2). For simplicity, these estimations assumed a random allele distribution in all loci and did not consider the very likely existence of yet undiscovered VNTR alleles.

**TABLE 2:** Metrics for each VNTR locus within the studied dataset, as inferred from MLVA typing.

**VNTR locus**	**Dye label**	**Repeat count range**	**Amplified flanks (bp)**	**PCR fragment size range (bp)**	**No. unique alleles**	**Simpson’s index of diversity**
SGPV_9	VIC	5–14	167–169^b^	272–463	10	0.821
SGPV_27	PET	3–4	339	375–387	2	0.501
SGPV_67^a^	6FAM	1–6	448	460–520	6	0.705
SGPV_143	NED	2–10	150	176–280	11	0.792
SGPV_177	PET	2–10	167	197–317	6	0.590
SGPV_218	VIC	2–6	449–451^b^	477–535	5	0.483
SGPV_221	NED	6–11	357	429–489	6	0.650
SGPV_227	6FAM	2–6	197	209–233	5	0.553

*^*a*^Two specimens lacking PCR amplicons corresponding to locus SGPV_67 are not considered here. ^*b*^Two single bp indels occurring in the flanks of both SGPV_9 and SGPV_218, respectively at up- and downstream positions relative to the repeats, cause the flank sizes of these loci to vary slightly across specimens.*

### MLVA Cluster Analysis and VNTR Stability

All MLVA profiles generated were imported into BioNumerics v7.6 (Applied Maths NV, Sint-Martens-Latem, Belgium), and Minimum-spanning-tree cluster analysis was performed with default settings. In the network thus resulting from comparison of the whole dataset, branches representing connection of profiles identical in ≤5/8 VNTR loci were hidden, a threshold deduced from the likelihood estimations outlined above, based on allelic diversity and dataset size. For Minimum-spanning-trees generated from smaller subsets of profiles, selected based on miscellaneous metadata (geographic origin etc.), branch connections of ≤4/8 identical loci were hidden.

As culturing of European SGPV variants has not yet been successful, the *in vitro* stability of the VNTR loci following repeated passages of the virus could not be assessed. The *in vivo* stability was however evaluated through typing of 16 gill samples harvested previously as part of a controlled SGPV infection trial. Moreover, comparison of likely epizootiologically linked samples collected over consecutive years from individual freshwater smolt farms suffering recurring SGPV outbreaks was also conducted, thus facilitating inference of the longer-term *in natura* stability of the targeted loci.

## Results

### Phylogenetic Analysis of SGPV Reveals a Transatlantic Divide in the Species

**FIGURE 2:**
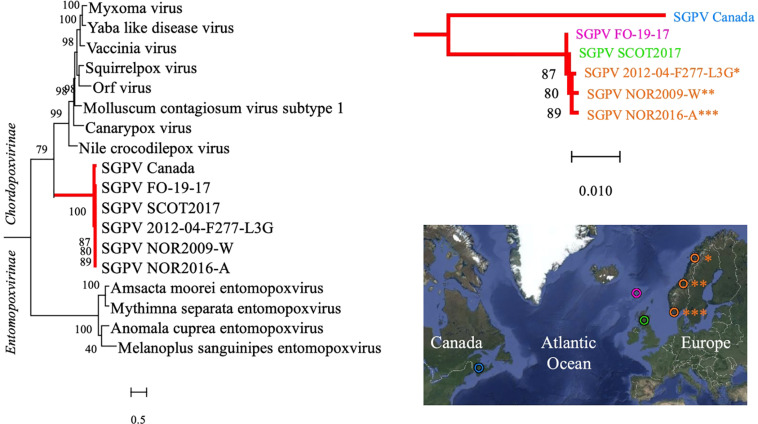
A Maximum Likelihood tree based on the concatenated amino acid sequences encoded by two genes (major capsid protein and DNA-directed RNA polymerase subunit beta) in various members of the *Poxviridae* family is shown on the left. The SGPV branch is further colored red and magnified on the upper right, illuminating its clear bifurcation into two distinct sub-lineages, respectively, comprising SGPV strains from Northeast America (Canada/blue) and Europe (Faroe Islands/pink, Scotland/green and Norway/orange), as shown in the map on the lower right. Sequences from the African swine fever virus (hidden) were included for tree rooting, and the aLRT SH-like method was employed for estimating branch support values. See Supplementary Figure S4 for trees based on individual alignment of the two genes and GenBank accession numbers. Map created with Google Maps (Map data/imagery: INEGI, Nasa, TerraMetrics).

Phylogeny inferred from the concatenated amino acid sequences encoded by two genes in selected *Poxviridae* species verified the findings of previous authors (Gjessing et al., 2015; Leblanc et al., 2019) that SGPV forms a deep, distinct and relatively conserved branch basally within the *Chordopoxvirinae* subfamily (Figure 2). Within the main SGPV lineage, a clear genetic separation could nevertheless be observed between the single specimens collected on the Atlantic coast of Canada and those of European origin. Trees built from alignment of the two genes individually shared compatible topologies (Supplementary Figure S4). Notably, as the published SGPV genome from Canada (inferred from transcriptome sequencing) is incomplete (Leblanc et al., 2019), inclusion of an extended panel of full-length genes for comparison was not possible.

### A Single-Tube Eight-Locus MLVA Enabling Specific, High-Resolution SGPV Genotyping

Approximately 90 putatively repetitive loci were identified from each of the five examined SGPV genomic contig sets. Following *in silico* and *in vitro* exclusion steps, eight VNTRs (designated SGPV_9, SGPV_27, SGPV_67, SGPV_143, SGPV_177, SGPV_218, SGPV_221, and SGPV_227), located at various positions throughout the publically available genome of the Norwegian SGPV strain 2012-04-F277-L3G (GenBank accession no. KT159937; Gjessing et al., 2015) (Supplementary Table S2), were ultimately selected for MLVA inclusion. While Sanger sequencing performed on 12 SGPV samples revealed minor repeat unit sequence heterogeneity in all loci but SGPV_218 and SGPV_227, repeat unit size uniformity was confirmed for all loci (data available upon request).

**FIGURE 3:**
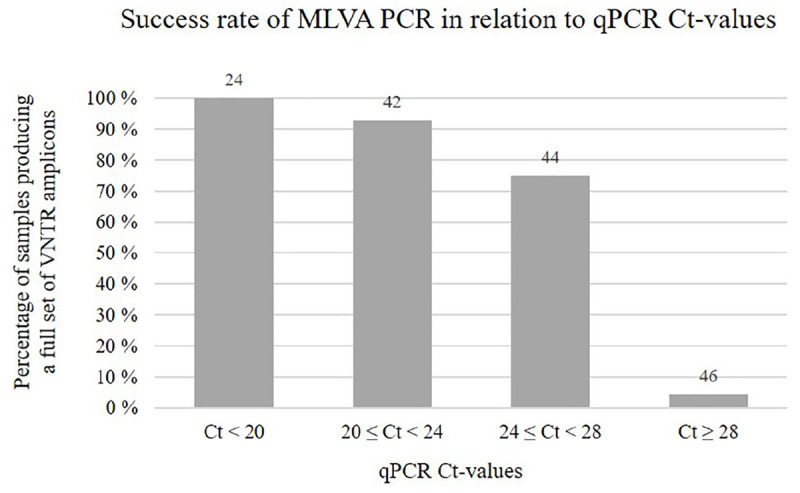
Chart showing the percentage of samples, within different qPCR Ct-value ranges, for which MLVA PCR produced a full set of eight VNTR amplicons. Numbers above the bars indicate the total number of samples falling within each range. Notably, for some of the samples MLVA typed in this study, qPCR Ct-value measures were not available.

A single-tube MLVA assay based on eight VNTR loci, involving multiplex PCR and capillary electrophoresis, was thus designed and tested. Upon running the final assay on 310 SGPV positive salmon gill samples, multiple specific PCR products were in most cases visually detectable as clear bands by gel electrophoresis (example in Supplementary Figure S1). From the remaining samples, only a few weak bands, or none at all, were observed, which was presumably due to the presence of insufficient amounts of SGPV template DNA for amplification of the targeted VNTR loci, as indicated by their relatively high Ct-values (Figure 3). In some cases, increasing the PCR template volume resolved this issue. Notably, no PCR amplicons were produced from controls consisting of SGPV negative (by qPCR) salmon gill samples.

Capillary electrophoresis was only attempted on samples showing PCR amplification of multiple VNTR targets by gel electrophoresis. Electrophoretic peaks following capillary electrophoresis, individually corresponding to either of the eight VNTR loci, could readily be distinguished based on fluorescent labeling and size (Supplementary Figure S2A). Size corrections according to inferred line-of-best-fit equations (Supplementary Figure S3) enabled accurate calculation of repeat counts. No size overlaps were recorded within the four pairs of loci labelled with identical dyes (Table 2).

While for most examined samples capillary electrophoresis revealed only eight fluorescent peaks (Supplementary Figure S2A), in other cases a higher number of peaks was observed (Supplementary Figures S2B–D), presumably due to the presence of more than one SGPV variant in these samples. Such cases could be easily resolved if this concerned only a single locus (Supplementary Figure S2B), but interpretation proved more problematic if multiple loci were affected. For some of these samples displaying ambiguity, qualified separation of VNTR amplicons belonging to the respective SGPV variants present could be performed based on the relative intensity of the fluorescent signals (Supplementary Figure S2C). In other cases this was not possible (Supplementary Figure S2D), however, and such samples were therefore not included in further analyses. For samples where separation of primary/secondary duplicate signals was possible (examples in Supplementary Figures S2B,C), two separate readings of eight electrophoretic peaks each were recorded and subjected to downstream analyses. Importantly, in such cases, both inferred profiles were often identified (together and/or individually) in several fish sampled from the same outbreak/farm/river, thus corroborating their authenticity. Throughout the dataset, ambiguities were observed multiple times in all eight loci.

From the 310 gill samples initially included in the study, 130 were thus excluded due to either lacking PCR amplification or unresolvable ambiguity following capillary electrophoresis. For the remaining 180 samples, 47 displayed duplicate signals, resulting in a total of 227 MLVA profiles recorded (Supplementary Table S1).

### Statistical Evaluation Exposes a Diverse Dataset and Projects Assay Robustness

The allelic diversity of individual VNTR loci within the examined dataset ranged from 2 to 11 alleles (excluding missing amplicons), with Simpson’s indexes of diversity ranging between 0.483 and 0.821 (Table 2). The Simpson’s index of diversity for full MLVA profiles (all eight loci combined) was 0.978. LIAN analysis identified a standardised index of association (*I_*A*_^*S*^*) of 0.0677 (*P*_Monte Carlo_ < 0.0001), which thus differs significantly from zero, confirming linkage disequilibrium.

Under the assumption of random allele distribution in all loci, and considering only the allelic diversity observed in this study (Table 2), the likelihood per MLVA profile for a chance match at 8/8 VNTR loci with at least one of the 226 other profiles examined, is 0.02%. Counting downward with regard to number of matching loci, the corresponding likelihoods are 0.8% (7/8 matches), 13.8% (6/8 matches), 77.4% (5/8 matches), and >99.9% (≤4/8 matches) (see Supplementary Datasheet), thus making individual MLVA connections between specimens represented by ≤5/8 identical loci untrustworthy. Considering the examined sample collection, however, these figures are presumably considerable overestimates – for one due to the likely existence of yet undiscovered alleles, but not least due to the inclusion of large sub-groups of epizootiologically related samples likely rendering the dataset less diverse than a truly random dataset of this size.

### MLVA Cluster Analysis Illuminates SGPV Population Dynamics

**FIGURE 4:**
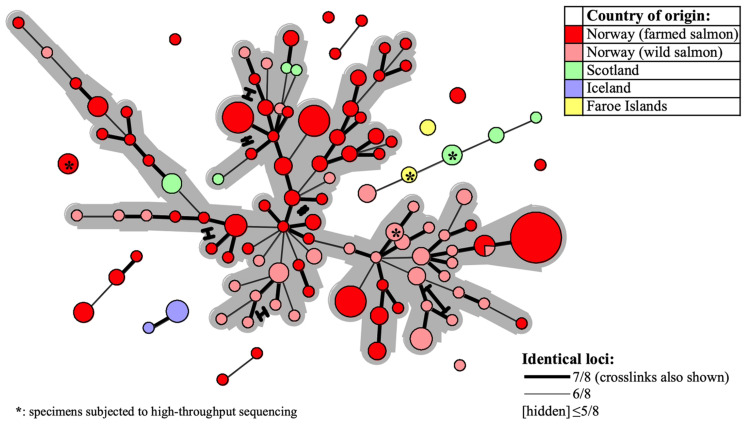
Minimum-spanning-tree based on 227 SGPV MLVA profiles detected from 180 Atlantic salmon gill samples in Europe (see Table 1 and Supplementary Table S1 for further details). Coloring is with regard to fish type and/or country of origin (see upper right legend) and specimens herein subjected to Illumina sequencing are indicated with asterisks. Declining MLVA similarity is indicated by the declining thickness of branch connections (see lower right legend). Alternative branch connections (crosslinks) have terminal demarcations. The continuous network of interconnections produced between 186 out of the 227 profiles, within a threshold of ≥6/8 identical loci, has a gray background.

For evaluation of putative epizootiological connections between the examined SGPV samples, MLVA cluster analysis was visualised in Minimum-spanning-trees. A continuous network of interconnections, dominated by Norwegian samples recovered from 1995 to 2019, linked 82% (186/227) of the MLVA profiles within a threshold of ≥6/8 identical loci (Figure 4). This proportion increased to 97 and 100%, respectively for ≥5/8 and ≥4/8 identical loci. Upon relating available information regarding time of sampling and/or outbreak severity (data not shown) to clustering within the network, no pervasive trends were observed.

**FIGURE 5:**
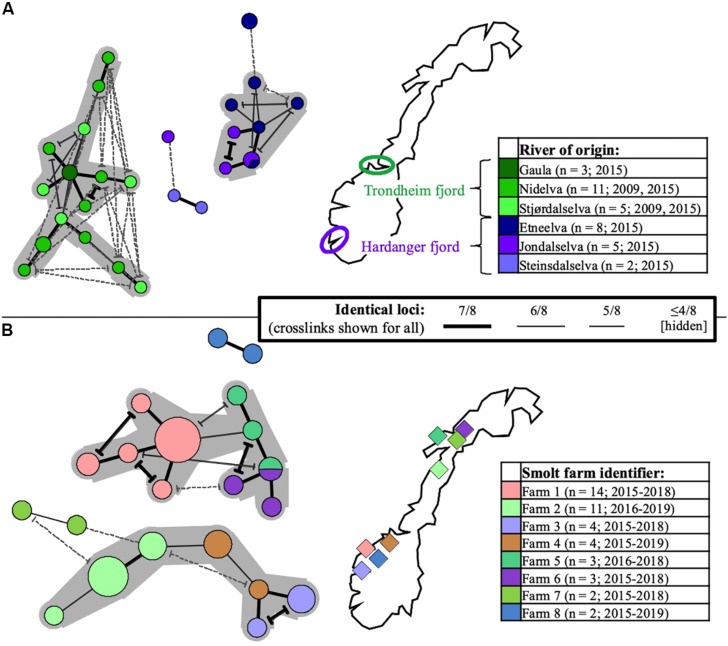
Minimum-spanning-trees based on SGPV MLVA profiles, with branch representations indicated in the common centered legend (alternative branch connections, or crosslinks, have terminal demarcations). Coloring is with regard to river- or smolt farm of origin in Norway (for reference and further details, see Supplementary Table S1), as indicated in separate maps/legends in the respective panels (number of profiles and sampling time span also shown). Larger groups of profiles interconnected through ≥ 6/8 identical loci are highlighted with a gray background. Panel **(A)** shows the relationships between 34 SGPV specimens originating from wild Atlantic salmon (returning spawners), respectively in three rivers emptying into the Trondheim fjord (shades of green) and three rivers emptying into the Hardanger fjord (shades of purple). Panel **(B)** shows the relationships between 43 SGPV specimens originating from eight different freshwater farms for Atlantic salmon smolt. Unless heterogeneity was observed, only single samples from individual case submission were included for comparison.

Particular sub-selections of specimens, for which a relaxed threshold of ≥5/8 identical loci was considered, displayed varying degrees of clustering bias linked to origin. Specifically, geographically linked clustering was documented through comparison of SGPV samples from returning wild salmon spawners in rivers connected to two distant fjord systems in Norway (Figure 5A). Furthermore, when comparing samples from Norwegian freshwater smolt farms, a similar trend was in several cases observed at the level of individual farms (Figure 5B). No strong, overarching geographic trends could be observed for this latter sub-selection, however, as the respective sub-lineages comprise specimens from both northern- and southern farms. Particularly noteworthy is the fact that while SGPV from farms 6 and 7 populate distinct MLVA sub-lineages, these two farms are located in the same fjord in northern Norway, a situation that is also mirrored by farms 1 and 8 in southern Norway. Only in some very few cases had wild- and farmed salmon been sampled in relative proximity to each other (see Figure 1), but in none of these cases was a high MLVA similarity observed across fish types.

**FIGURE 6:**
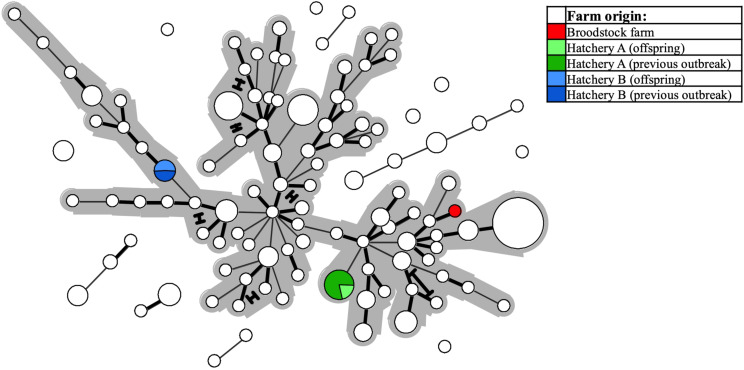
The same Minimum-spanning-tree as presented in Figure 4, but with coloring (see legend) here reflecting specific farm origins – i.e., from a single Atlantic salmon broodstock farm, or from either of two hatcheries, both of which experienced SGPV disease outbreaks in offspring from said broodstock farm, and also in thus unrelated, noncontact fish groups.

Two investigated cases involved suspicion of vertical SGPV transmission from a single Atlantic salmon broodstock farm to two separate recipient hatcheries, as SGPV disease outbreaks were diagnosed at the juvenile stage in both sibling groups. SGPV positive samples for comparison were also available from said broodstock farm, and from recent SGPV disease outbreaks having occurred in separate (unrelated) noncontact fish groups at both hatcheries. The Minimum-spanning-tree based on the entire dataset reveals that none of the offspring batches harbored SGPV variants similar to that found in the parental broodstock – instead, these formed perfect MLVA matches with the specimens found previously in the respective hatcheries (Figure 6).

Of note, typing of 16 gill samples harvested from individual fish following a previous SGPV infection trial revealed complete VNTR homogeneity amongst the resulting MLVA profiles, which also matched perfectly with that of the challenge strain.

## Discussion

The capacity to discern genetic relationships between conspecific specimens of a pathogen with a high resolution is central for resolving, e.g., transmission events underlying their dissemination. Barring available culturing methods for SGPV, we developed a single-tube eight-locus MLVA assay that enables specific, high-resolution genotyping of this virus directly from infected gill tissue. Initial phylogenetic analysis verified a transatlantic genetic divide in this species, while MLVA typing and cluster analysis performed on samples from infected Atlantic salmon allowed interpretation of SGPV population dynamics in Europe.

It was recently documented that SGPV is widespread amongst wild populations of Atlantic salmon along the Norwegian coast (Garseth et al., 2018), and detections of the virus in historic gill samples from farmed salmon verifies that its presence in Norway dates back at least two decades. Moreover, while the overall prevalence of SGPV in wild- and farmed salmon across the Northeast- and Subarctic Atlantic Ocean remains largely unknown, its presence has been confirmed in several countries throughout this region. Phylogenetic analysis performed here revealed a clear transatlantic separation amongst SGPV specimens from Norway (*n* = 3), Scotland (*n* = 1), the Faroe Islands (*n* = 1), and Canada (*n* = 1), which also shows a highly conserved “European” lineage (Figure 2). The MLVA assay further proved applicable for a diverse collection of SGPV specimens from Europe (Norway, Scotland, Faroe Islands, and Iceland), but none of the targeted loci could be detected *in silico* in the published (incomplete) SGPV genome from the east coast of Canada (Leblanc et al., 2019). Pending publication of a full SGPV genome from this region, the capability of the present assay for genotyping SGPV variants outwith the European lineage therefore remains uncertain. The present lack of comprehensive methods allowing cultivation and isolation of SGPV in pure culture will further likely represent a significant obstacle for any high-resolution genotyping effort aimed at this virus. Some samples thus proved unsuitable for MLVA typing due to either low viral loads (Figure 3) or apparent multi-strain infections (Supplementary Figure S2).

A Simpson’s index of diversity of 0.978 for all eight targeted VNTR loci combined reflects the high-resolution typing made possible by the assay, while complete VNTR homogeneity across all samples from a single controlled infection trial assures assay robustness and sufficient stability for epizootiological relevance. Although the observation of ambiguous MLVA signals prompted the exclusion of some samples from the study, two separate MLVA readings could often be reliably inferred from individual samples (Supplementary Figure S2). In such cases, both detected profiles commonly recurred (together and/or individually) in several fish from the same site. It is therefore reasonable to presume that this reflects a scenario involving the preceding presence of multiple SGPV variants in said environments, rather than spontaneous mutations arising in the virus after infection.

Although phylogenetic analysis points to one main, relatively conserved, SGPV lineage dominating in the Northeast Atlantic region, European specimens populate dispersed and partly disconnected MLVA clusters (Figure 4). This also concerns the four samples herein subjected to Illumina sequencing. Most of these closely related MLVA sub-lineages are, however, non-exclusive in terms of source countries, which could indicate one or more shared historic SGPV reservoir(s) for this whole region. Of note, while SGPV samples typed from a single location in Iceland do not interconnect with any other European specimen under the employed threshold, it may be that the typing of an extended panel of Icelandic samples would have resulted in such connections. Based on available clinical information from the investigated cases, clustering neither provides any clues for assuming that specific sub-lineages of the virus are intrinsically more or less virulent. It thus appears that differences in disease presentation, which are sometimes observed across consecutive SGPV detections within individual salmon farms, are more likely due to external factors relating to the fish and/or environment, although spontaneous mutational changes in the virus may also conceivably affect virulence. In this regard, genetic recombination (e.g., causing gain, loss or expansion of virulence genes) has since long been recognized as a readily occurring evolutionary mechanism in various other poxviruses (e.g., Fenner and Comben, 1958; Bedson and Dumbell, 1964; Pickup et al., 1984; Esposito et al., 2006; Elde et al., 2012; Qin and Evans, 2014). While the prevalence of recombination in SGPV remains uncharted, and although linkage disequilibrium was confirmed for the studied loci and samples, the relatively low *I_*A*_^*S*^* (0.0677) is comparable to that previously estimated for multi-locus genotyping data from a diverse collection of the highly recombinant bacterium *Neisseria meningitidis* (Feil et al., 1999; Haubold and Hudson, 2000).

Despite what presents as an overall lack of distantly separated genetic lineages within the sampled SGPV population, scrutiny of restricted sub-selections of samples serve to exemplify the inherent potential of the MLVA assay for fine-scale epizootiological inference. For instance, discrete clustering was observed for SGPV specimens from wild, returning salmon spawners migrating through separate Norwegian fjord-systems, but only minor colocation bias occurred at the level of individual rivers (Figure 5A). For the Trondheim fjord, the co-clustering notably prevails despite inclusion of samples collected six years apart (2009 and 2015). While not ruling out the possibility for riverine spread, these observations are consistent with a situation involving at least some degree of marine, inshore SGPV transmission, originating either from other wild salmon homing for neighboring rivers, or from alternative reservoirs (e.g., infected farmed salmon; see below) in this temporarily shared environment. This scenario also adheres to the lack of SGPV detections in wild, non-anadromous (landlocked) populations of Atlantic salmon in Norway (Garseth et al., 2018; Gåsnes et al., 2019).

These geographic trends are not consistent for all Norwegian rivers represented in the study, however, as near-identical MLVA profiles were also occasionally detected in wild-fish samples from distant regions. Interpretation of this is complicated by the nationwide occurrence of this virus in Norwegian salmon aquaculture, which represents a potential infection reservoir for wild salmon (and vice versa), and where no overarching geographic trends could be inferred from MLVA clustering (example in Figure 5B). The observation of SGPV variants that appear geographically relatively unrestricted, likely reflects historic instances of remote viral spread having contributed toward obscuring any pre-existing local signals. This may, e.g., have happened through anthropogenic activities (transport of infected fish or roe/milt), or offshore encounters with transmission between migrating wild salmon. The latter explanation currently seem less likely though, both due to a low offshore host density, and the present lack of SGPV detections in wild salmon at sea (Gåsnes et al., 2019). Information regarding potential non-salmon reservoirs is also lacking. Notably, while the few samples collected from wild- and farmed salmon in nearby areas displayed relatively low MLVA similarities, these observations do not provide sufficient basis for making firm predictions regarding the extent of transmission occurring between wild- and farmed fish. These matters could be the focus of future investigations.

At the level of individual freshwater farms for salmon smolt in Norway, there is nevertheless a tendency for consecutive SGPV outbreaks over multiple years and generations of fish being caused by specific sub-lineages of the virus (Figure 5B). Likely, this either reflects farms with persistent SGPV “house-strains”, or alternatively repeated introductions from specific source reservoirs (e.g., via intake fresh-/seawater or infected juvenile fish/eggs). Notably in this latter regard, however, Norwegian regulations require disinfection of all intake water to such facilities unless collected from sources free from anadromous fish. While the modes by which these putative SGPV “house strains” emerge and recur in farms may vary and remains open for discussion, it is worth mentioning that one of the heavily affected smolt farms included in this study did attempt extended disinfection of its facilities. The consecutive fish batch was then documented SGPV negative by qPCR screening through all early life stages, but experienced an SGPV outbreak shortly after intraperitoneal vaccination. An MLVA profile previously exclusive to that farm was confirmed in gill samples from both diseased and clinically unaffected fish. Thus, while the disinfection attempt appeared successful initially, the technologically complex vaccination machine (not included in the disinfection protocol) likely preserved the virus at the farm and also served as a direct source of infection. Furthermore, although anecdotal speculations of a vertical transmission route for SGPV have been proposed, no indication of this was found in the present study by investigating two cases involving such suspected circumstances. Conversely, in both these particular instances, MLVA typing refuted an epizootiological link between the parental broodstock and the parallel offspring groups, and instead provided strong support for re-appearing SGPV “house strains” at the respective freshwater landing farms as the source of infection (Figure 6). These mentioned real-life applications of the developed MLVA assay highlight its potential as an expedient investigative tool for rapid tracing of SGPV detections.

## Conclusion

A single-tube eight-locus MLVA genotyping scheme was developed and employed for typing of a large collection of SGPV samples from farmed and wild Atlantic salmon in Northern Europe. Phylogenetic analysis in combination with MLVA typing point to one main SGPV lineage as dominating in this region, with a distinct variant previously detected on the Canadian Atlantic coast. Further scrutiny, by MLVA, of smaller sample groups from discrete origins enabled identification of specific SGPV sub-lineages likely reflecting locally predominant viral strains within fjord systems, as well as persistent and/or recurring “house strains” in freshwater smolt farms. The assay was also used to disprove suspected vertical transmissions of SGPV and to verify a failed disinfection attempt, thus emphasizing its potential application for inferring infection routes and during evaluation of ameliorative measures. No indication of intrinsically high- or low-virulent SGPV sub-lineages was found. In summary, the method represents a highly sensitive and easily implemented tool that is well suited for molecular tracing of SGPV detections.

## Data Availability Statement

Assembled contigs of the four SGPV samples subjected to Illumina sequencing in this study will be made available upon request. Major capsid protein and DNA-directed RNA polymerase subunit beta gene sequences, extracted from the contig sets, have been deposited in NCBI GenBank under accession numbers MT165477–MT165484. See also Supplementary Material.

## Ethics Statement

The animal experiment referred to in this study was approved by The Norwegian Animal Research Authority (FOTS ID: 15042) and conducted in accordance with the European Union Directive 2010/63/EU for animal experiments.

## Author Contributions

SG, MD, TT, MG, and OD contributed to the conception and design of the study. TT filtered and assembled sequence data. SG performed all further *in silico* analyses. SG, MD, and SM planned and carried out all *in vitro* procedures. MG, ÅG, KS, TM, PP, and BT acquired samples and crucial metadata for the study. SG drafted the manuscript, with input from MD and TT. All authors later contributed critical input through further revisions.

## Conflict of Interest

The authors declare that the research was conducted in the absence of any commercial or financial relationships that could be construed as a potential conflict of interest.
