# Supplementary figures and images for: Genotyping of Salmon Gill Poxvirus Reveals One Main Predominant Lineage in Europe, Featuring Fjord- and Fish Farm-Specific Sub-Lineages
Source: Front Microbiol. 2020 May 29;11:1071. doi: 10.3389/fmicb.2020.01071 (PMC7272583; doi:10.3389/fmicb.2020.01071)

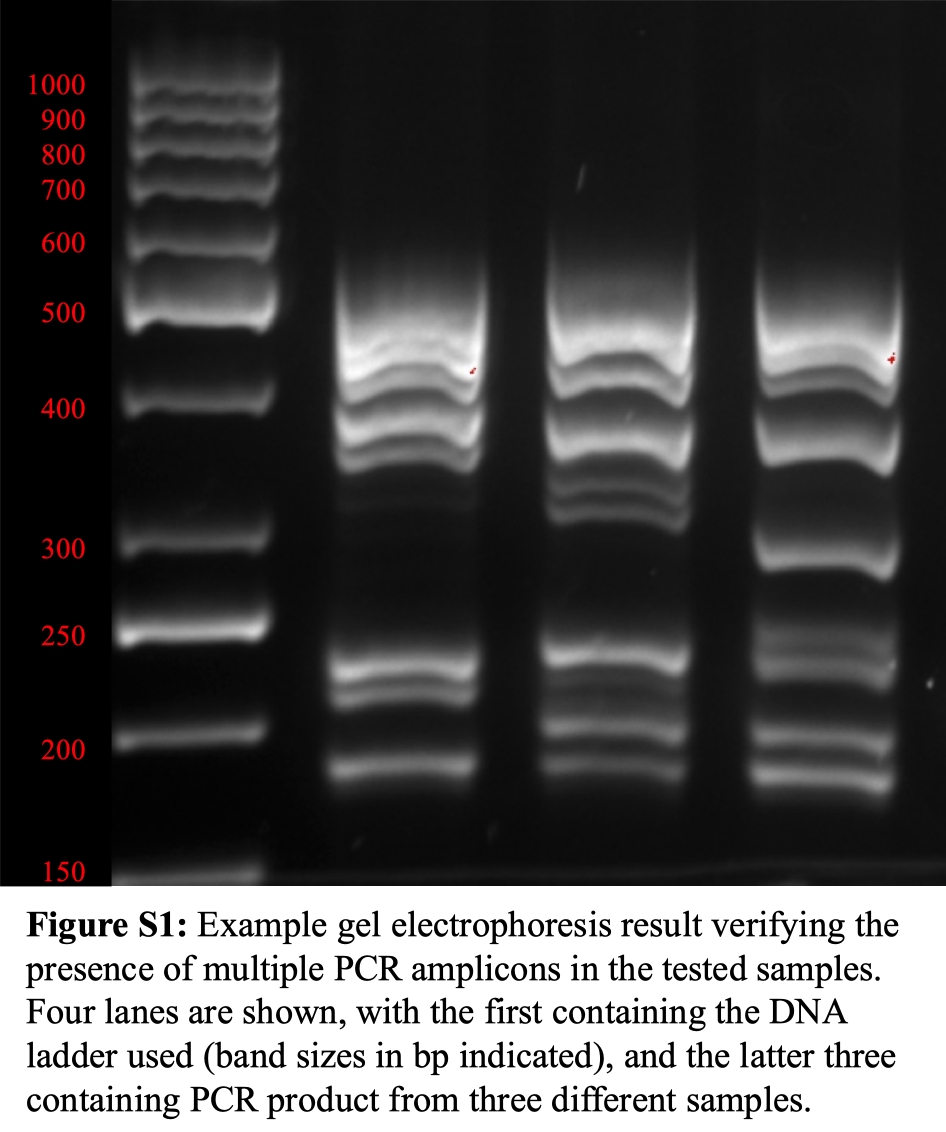

Supplement: Supplementary file 1 [file Image_1.JPEG]

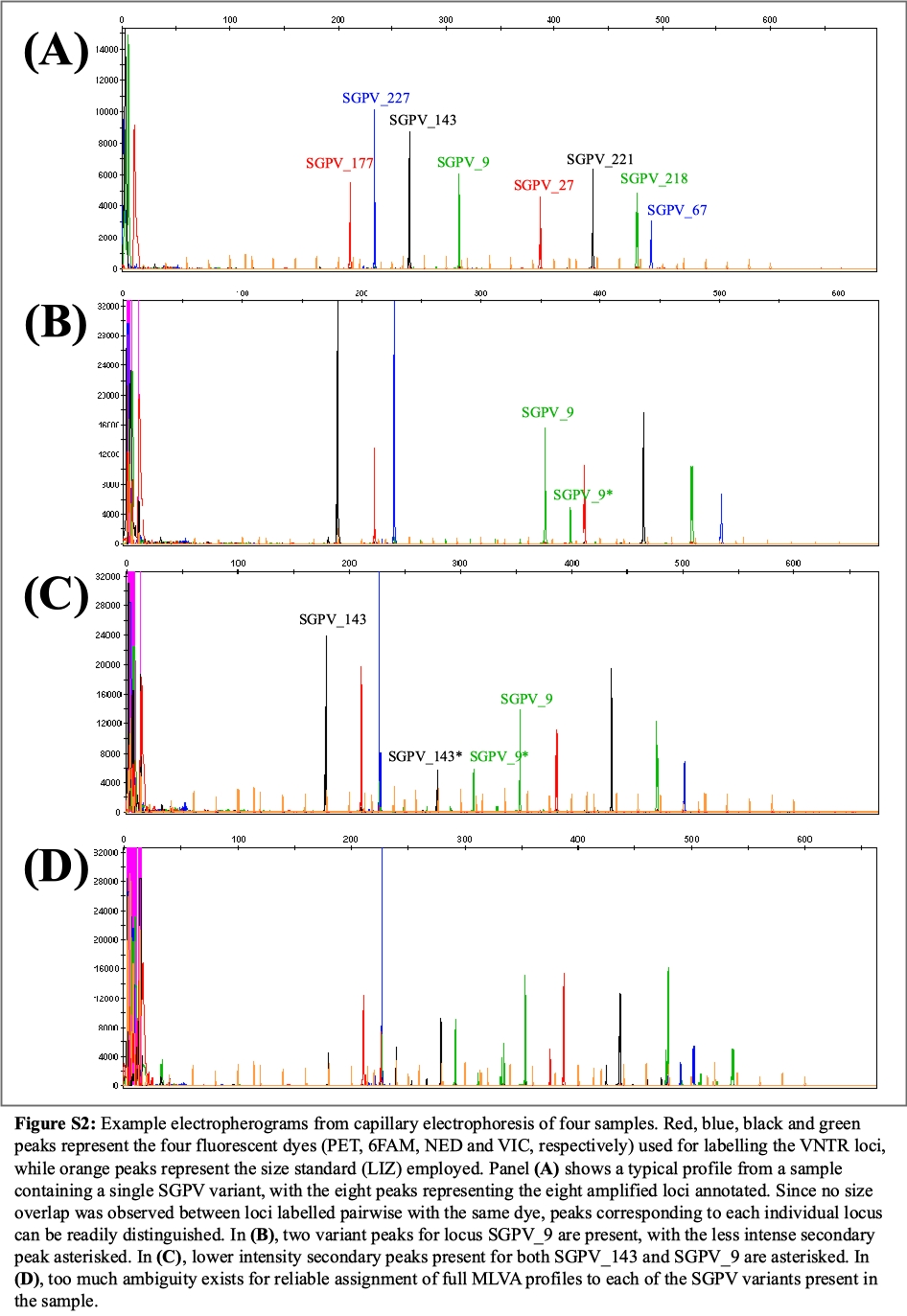

Supplement: Supplementary file 2 [file Image_2.JPEG]

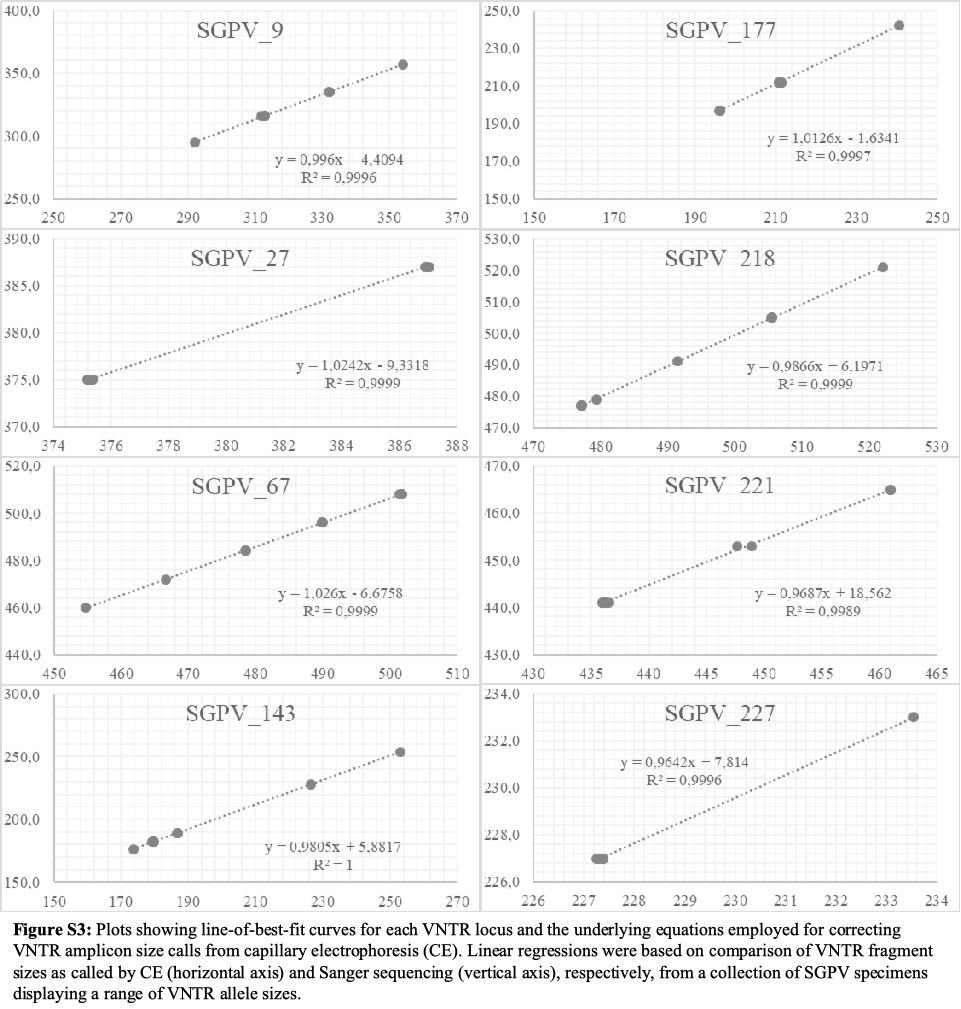

Supplement: Supplementary file 3 [file Image_3.JPEG]

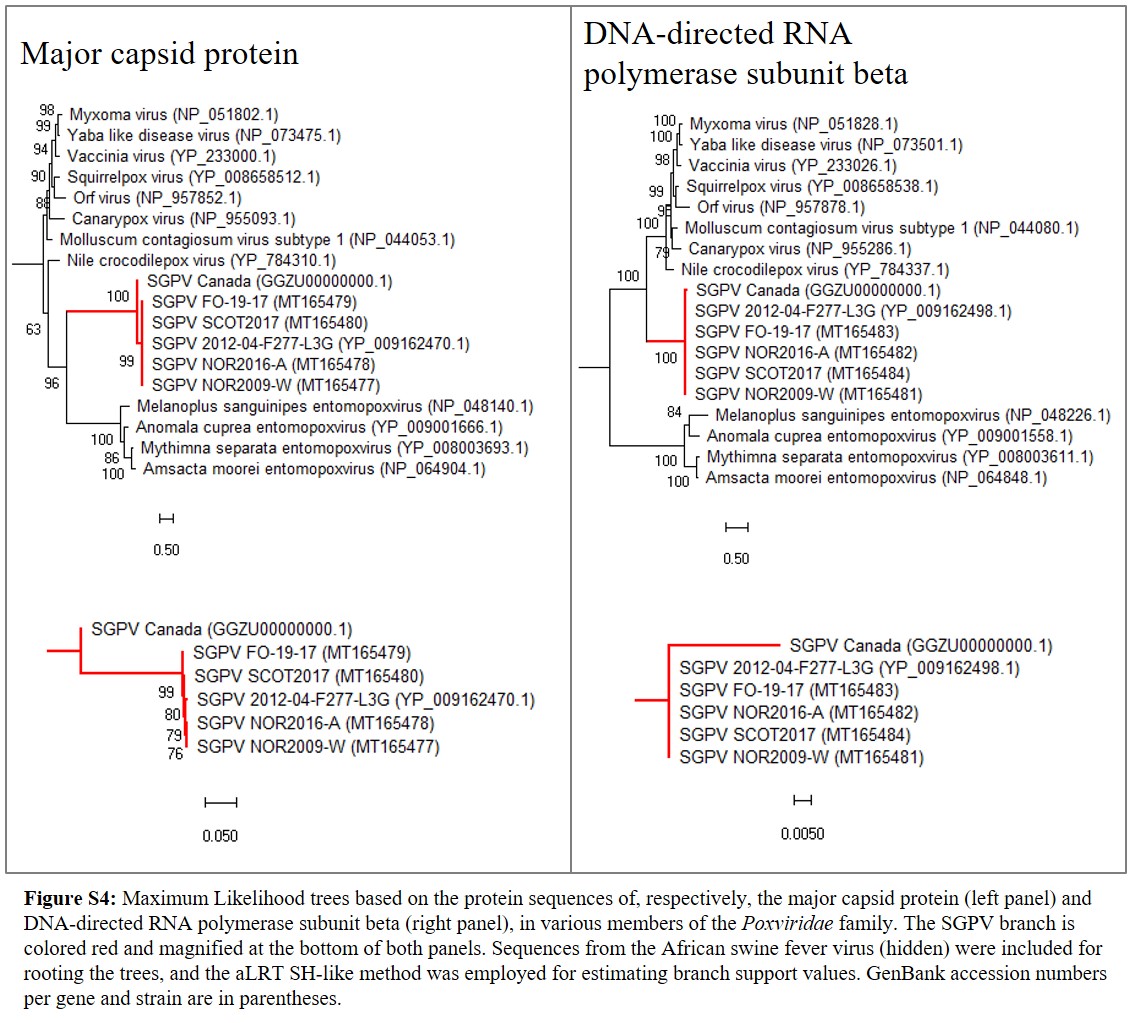

Supplement: Supplementary file 4 [file Image_4.jpg]
