# Supplementary material for: Genotyping of Salmon Gill Poxvirus Reveals One Main Predominant Lineage in Europe, Featuring Fjord- and Fish Farm-Specific Sub-Lineages
Source: Front Microbiol. 2020 May 29;11:1071. doi: 10.3389/fmicb.2020.01071 (PMC7272583; doi:10.3389/fmicb.2020.01071)
Supplement: Supplementary file 6 [file Table_2.DOCX]

| **Table S2:** Characteristics of the selected VNTR loci and surrounding genetic content within the genome of strain 2012-04-F277-L3G (GenBank accession no. KT159937). Protein annotations are with reference to Gjessing *et al.* (2015). | | | |
| --- | --- | --- | --- |
| **VNTR locus** | **Genomic location (bp span)** | **Surrounding protein-coding gene(s) (GenBank accession no. / gene no.)** | **Repeat unit (size in bp)** |
| SGPV_9 | 9070-9258 | Within hypothetical type I transmembrane helix protein (AKR04133 / SGPV009) | AATGAAATTACTAAGTTATAC (21) |
| SGPV_27 | 26555-26602 | Within hypothetical type I transmembrane helix protein (AKR04155 / SGPV031) | TCATCAGAGAAG (12) |
| SGPV_67 | 66584-66643 | Within hypothetical protein (AKR04199 / SGPV075) | CCAGGAGGAGTA (12) |
| SGPV_143 | 142905-142930 | Between RING-finger-containing E3 ubiquitin ligase (AKR04263 / SGPV139) and mRNA capping enzyme, small subunit (AKR04264 / SGPV140) | ATCATGTAATAAT (13) |
| SGPV_177 | 176573-176602 | Within hypothetical secreted protein (AKR04285 / SGPV161) | CCTGATGACCCAAAG (15) |
| SGPV_218 | 218280-218321 | Between two hypothetical proteins (AKR04312 / SGPV188 and AKR04313 / SGPV189) | ATATTAAAAAATGA (14) |
| SGPV_221 | 221091-221174 | Within hypothetical protein (AKR04315 / SGPV191) | TCTTGACAGTGA (12) |
| SGPV_227 | 227355-227384 | Within hypothetical protein (AKR04322 / SGPV198) | GACACA (6) |
