# Supplementary material for: Genotyping of Salmon Gill Poxvirus Reveals One Main Predominant Lineage in Europe, Featuring Fjord- and Fish Farm-Specific Sub-Lineages
Source: Front Microbiol. 2020 May 29;11:1071. doi: 10.3389/fmicb.2020.01071 (PMC7272583; doi:10.3389/fmicb.2020.01071)
Supplement: Supplementary file 7 [file Table_3.DOCX]

| **Table S3:** Primer sequences used for multiplex PCR of VNTR loci. The fluorescent dye label (6FAM, VIC, NED or PET) used at the 5′ end of either forward or reverse primers are in **bold**. | | |
| --- | --- | --- |
| **VNTR locus** | **Forward primer (5′→ 3′)** | **Reverse primer (5′→ 3′)** |
| SGPV_9 | **VIC-**TTACCTATTACACAGAACGCTCGG | ATAAGGTATCTTTCGGAGATTGTCC |
| SGPV_27 | **PET-**TCACCAAGGTAACAACCAAGATGA | CGGTTCAACTTCCAAAGGGTATTC |
| SGPV_67 | **6FAM-**GTGATCATGTTGTGTGTTCAGCAA | TGTTGGCTTCAGGAAAACTAAGAC |
| SGPV_143 | CCAGAGAGCATCGAGTTCCATATT | **NED-**TTCTAGAAGAAGACCTACCCGAGT |
| SGPV_177 | **PET-**AGAAAGAGAGACAGTGCTGATTCT | AGATTCAATGAACTGTACACGGTG |
| SGPV_218 | CGGTCAAATAAAAACATCCCCGTT | **VIC-**CCGTGGTCCTGTGATGTAAAAATT |
| SGPV_221 | **NED-**AGGTCAGGAATTTACAAACGATGG | ATCAACGTACCGAAAGAAGGATGG |
| SGPV_227 | ACTCAGTAAAACAACGTCCAAATGG | **6FAM-**ACGTGTATTCTGAGAGTATCGGAC |
